# Ecological assessment of physico-chemical factors influencing the diversity and abundance of cyanobacteria in lakes of Côte d’Ivoire (Kan, Koubi, Loka, and Tiebissou)

**DOI:** 10.1093/femsec/fiag035

**Published:** 2026-03-31

**Authors:** Soumahoro Fatoumata Grace, Aw Sadat, Yao Djeha Rosine, Ouattara Bamory, Kouadio Meliton Djezou, Coulibaly Kalpy Julien

**Affiliations:** Institut National Polytechnique Félix Houphouët-Boigny de Yamoussoukro, BP 1093, Yamoussoukro, Côte d’Ivoire; Département Environnement Santé/ Unité de Chimie et de Microbiologie Environnementale, Institut Pasteur, BP 490, Abidjan 22, Côte d’Ivoire; Institut National Polytechnique Félix Houphouët-Boigny de Yamoussoukro, BP 1093, Yamoussoukro, Côte d’Ivoire; Société de Distribution d’Eau de la Côte d’Ivoire, (SODECI) 01, BP 1843, Abidjan 01, Côte d'Ivoire; Département Environnement Santé/ Unité de Chimie et de Microbiologie Environnementale, Institut Pasteur, BP 490, Abidjan 22, Côte d’Ivoire; UFR Biosciences, Université Félix Houphouët-Boigny de Cocody, 22 BP 582, Abidjan 22, Côte d’Ivoire; UFR Biosciences, Université Félix Houphouët-Boigny de Cocody, 22 BP 582, Abidjan 22, Côte d’Ivoire; Département Environnement Santé/ Unité de Chimie et de Microbiologie Environnementale, Institut Pasteur, BP 490, Abidjan 22, Côte d’Ivoire

**Keywords:** chlorophyll-a, cyanobacteria community, lakes, physico-chemical properties, Côte d’Ivoire

## Abstract

Cyanobacteria are photosynthetic microorganisms found in all aquatic ecosystems. Some species produce toxins, posing risks to human and animal health. In Côte d’Ivoire, information on the composition and dynamics of freshwater cyanobacterial communities remains limited. This study aims to assess the influence of physico-chemical parameters on alpha diversity, cyanobacterial abundance, and phytoplankton biomass, while comparing community responses to anthropogenic pressures (urban, agricultural, and aquaculture). Nine sampling campaigns conducted between March 2023 and May 2024 included in situ measurements (pH, temperature, and turbidity) and laboratory analyses of nutrients and chlorophyll-a. Cyanobacteria were identified microscopically and quantified using a Malassez chamber. A total of 19 genera were recorded. Highly impacted lakes were dominated by opportunistic taxa (Aphanocapsa, Pseudanabaena, and Microcystis), whereas the reference lake was characterized by genera with high ecological plasticity (Cylindrospermopsis and Anabaena). Cyanobacterial abundance was positively correlated with pH and temperature, chlorophyll-a with pH and turbidity, and alpha diversity declined with increasing turbidity. Multivariate analyses (GLM, RDA, and NMDS) confirmed a significant influence of environmental variables on community structure. These findings indicate that cyanobacterial dynamics are primarily driven by physicochemical conditions and the intensity of anthropogenic pressures, highlighting the need for targeted ecological monitoring to support sustainable freshwater management.

## Teaser

Thawing of ancient Siberian permafrost under controlled temperature conditions in NO and N-supplemented microcosms showed N_2_O accumulation, and metagenome-assembled genomes revealed that N emissions were driven by incomplete denitrifying bacteria.

## Introduction

Freshwater is a valuable but limited resource, unevenly distributed across the planet, with only a small fraction readily available for human use, as the majority is stored in glaciers, ice caps, or deep groundwater reservoirs. Among the freshwater resources accessible to humans, surface waters, including rivers, lakes, and reservoirs, play a crucial role both for human activities and for the maintenance of aquatic ecosystems (Morel 2007, Zhou and Zhao 2011, Vasistha and Ganguly 2022).

Lakes, whether natural or artificial, represent an important component of surface waters and constitute a major source of water for domestic, agricultural, and industrial purposes worldwide (Vasistha and Ganguly 2022). However, rapid population growth continues to exert increasing pressure on these resources, leading to water quality degradation and disrupting the functioning of aquatic ecosystems.

According to the World Health Organization (WHO), nearly two-thirds of the global population may face water stress by 2050, primarily due to the overexploitation of water resources for agriculture and food production (Islam and Karim 2019, Panhwar 2022). The preservation of surface water reservoirs is therefore critical, as they provide essential ecosystem services such as potable water supply, agricultural irrigation, recreational and tourism activities, while also serving as vital habitats for aquatic biodiversity (Subramaniam et al. 2023, Syeed et al. 2023). Consequently, the deterioration of water quality whether caused by eutrophication or other forms of pollution has become a major global issue, both for public health and for the sustainable management of water resources (Dixit and Shrivastava 2013, Syeed et al. 2023).

These aquatic ecosystems are continuously enriched with nutrients, particularly phosphorus and nitrogen, resulting from domestic, industrial, and agricultural discharges. This process fosters the progressive eutrophication of water bodies, which is generally marked by massive and recurrent blooms of cyanobacteria (Merhabi et al. 2019, Abahi et al. 2023).

Cyanobacterial blooms in surface waters significantly impair both raw and treated water quality. They alter the physical and chemical characteristics of the resource by changing its color, odor, and taste, and by increasing turbidity and pH levels (Quer et al. 2024). When these blooms involve toxin-producing species, they may pose serious health risks. Indeed, certain cyanobacteria are capable of producing toxins that are harmful to both human and animal health (Casero et al. 2019, Zerrifi et al. 2021, Ricciardelli et al. 2023). These compounds are largely resistant to conventional water treatment methods and may persist in treated water (Warren et al. 2010, Jalili 2022). As a result, some water sources affected by large-scale cyanobacterial blooms are occasionally abandoned due to limitations in treatment efficacy and the associated costs (Piontek et al. 2023). Such situations can rapidly lead to drinking water crises, especially in regions that rely exclusively on surface water sources. It should be noted that the abundance of cyanobacteria and chlorophyll-a concentrations are strongly influenced by multiple environmental factors (Zhao and Huang 2014). Elevated nutrient levels, particularly nitrogen and phosphorus, are recognized as key factors influencing cyanobacterial growth. High temperatures affect cyanobacterial communities by favoring thermophilic species while reducing the abundance of more sensitive ones. Additionally, factors such as dissolved oxygen, conductivity, and turbidity influence species composition and physiological processes. Water transparency and turbidity also modulate light penetration, which in turn regulates photosynthetic activity (Jankowiak et al. 2019).

The central region of Côte d’Ivoire is a major agricultural area, characterized by high population abundance and ongoing industrial development. As a result, many water bodies are located in urban areas and are subject to significant anthropogenic pressures, both urban and industrial. Despite this, these water bodies remain the primary sources of drinking water for the region. Lakes Kan, Loka, the Tiébissou reservoir, and Lake Koubi are essential supply sources for the local population, although they are not immune to environmental degradation and ecosystem disturbances (Wandan and Zabik 1996).

Various human activities have developed around these water bodies. Artisanal fishing is practiced, and certain reservoirs, such as Lake Koubi, host aquaculture operations, including floating cage fish farms. Agricultural irrigation is another major activity in the region. The shores of these reservoirs are heavily exploited for market gardening, and water is also used to irrigate staple crops such as cassava and yam, thereby contributing to local food security. However, these activities exert considerable pressure on aquatic communities. Intensive water use for irrigation, combined with nutrient inputs from fertilizers and agricultural runoff, promotes eutrophication, leading to the excessive growth of microalgae and cyanobacteria. Moreover, unregulated artisanal fishing can disrupt fish populations and alter the trophic structure of lakes, thereby impacting biodiversity and ecological balance. These disturbances may reduce ecosystem resilience to anthropogenic pressures and compromise the quality of water intended for human consumption.

Despite the strategic importance of these water resources, studies on the composition and dynamics of cyanobacterial communities in this region remain scarce. Accurate identification of the species present is essential for assessing and managing the risks associated with their proliferation. While cyanobacteria are widely distributed around the world and play a major ecological role, knowledge of their freshwater communities in Côte d’Ivoire remains limited. The first records of these microorganisms in the country date back to the 1960s, with studies by (Bourrelly 1961, Uherkovich and Rai 1977). More recent studies, including those by (Da 1992, Ouattara 2000), (Kouassi et al. 2016), suggest that the diversity of Ivorian cyanobacteria has been largely underestimated in earlier literature. Therefore, the collection of new data from various natural habitats across the country is essential to better understand their diversity.

This study represents the first comprehensive ecological assessment of cyanobacterial communities in four freshwater lakes located in central Côte d’Ivoire (Kan, Loka, Koubi, and Tiébissou). It aims to evaluate the influence of physicochemical parameters on alpha diversity, cyanobacterial abundance, and phytoplankton biomass, while comparing the ecological responses of these communities to different types of anthropogenic pressures (urban, agricultural, and aquaculture). The overarching goal is to identify the key environmental drivers controlling cyanobacterial proliferation in reservoirs used for drinking water supply.

## Material and methods

### Study areas and environmental setting

**Figure 1:**
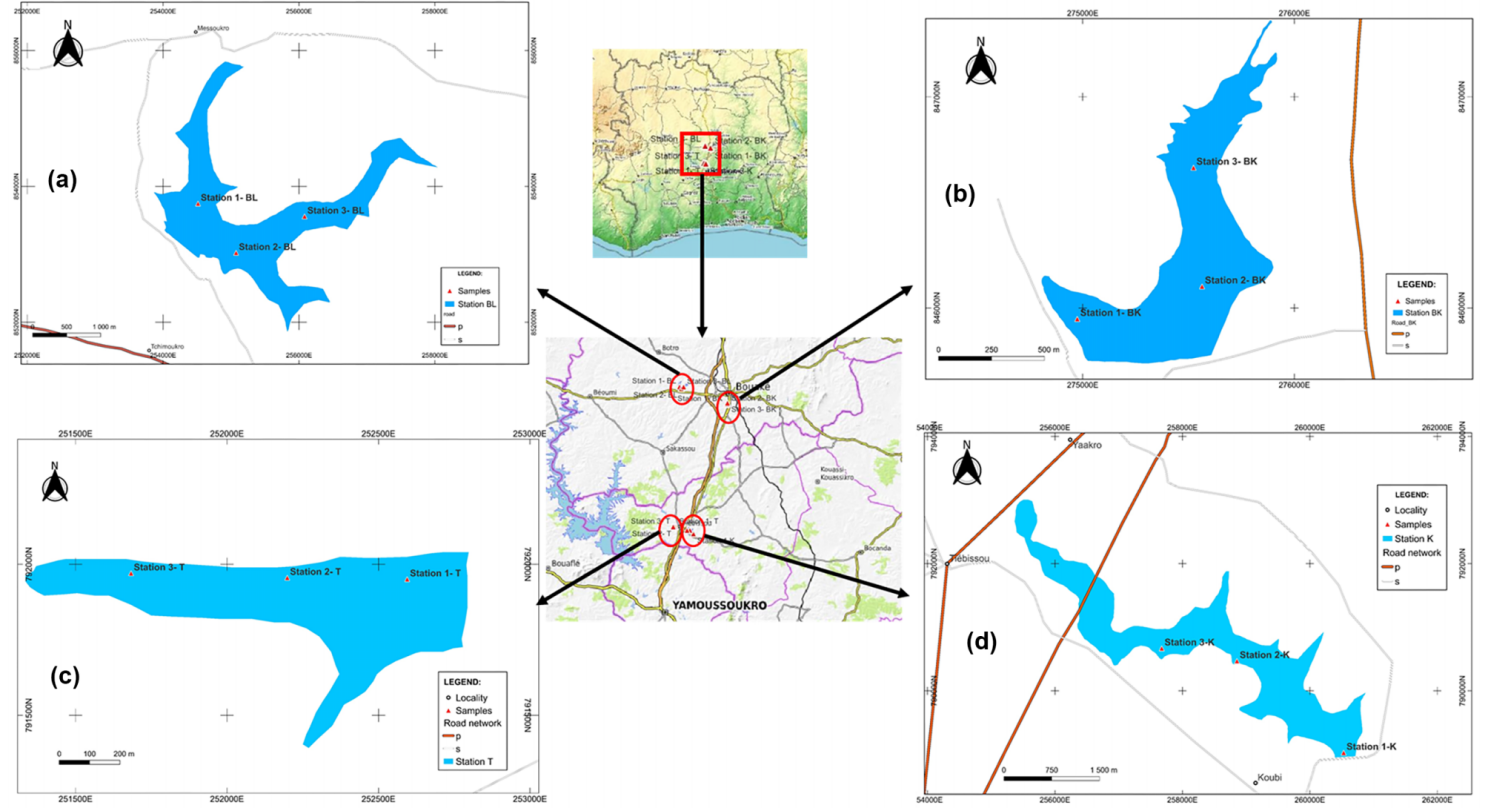
Study area and sampling stations (a) Lake Loka (Bouaké); (b) Lake Kan (Bouaké); (d) Lake Koubi (Tiébissou); (c) Lake Tiebissou. The numbers 1, 2, 3 preceding the station codes (K, T, BL, and BK) indicate the sampling point number within each lake.

This study was conducted on four freshwater bodies Kan, Loka, Koubi, and the Tiébissou reservoir, located in the Gbêkê and Bélier regions of central Côte d’Ivoire (Fig. 1). The study area lies between 6°55′12″ and 7°41′37.9″ north latitude and between 5°03′00″ and 5°49′60″ west longitude. It is characterized by a tropical savanna climate with two distinct seasons: a rainy season from April to November and a dry season from December to March. Annual rainfall ranges from 1200 to 1300 mm, with an average annual temperature of approximately 26°C (Koffi et al. 2023). Vegetation is dominated by grassy savannas interspersed with scattered shrubs and trees, typical of humid tropical and Sudanian savannas. The hydrographic network is mainly associated with the Bandama River and its tributaries, along with numerous secondary and seasonal watercourses (Zro et al. 2014, Yao et al. 2023).

The four lakes were selected to represent varying types and degrees of anthropogenic disturbance, while also serving as sources of drinking water for local communities. Lake Kan, located in the heart of Bouaké, is an urban water body surrounded by residences, vegetable gardens, and recreational areas, receiving both domestic effluents and urban stormwater. The Loka Lake, a relatively well-preserved peri-urban water body, is subject to minimal anthropogenic pressures and serves as a reference site for ecological comparisons. In the Bélier region, Lake Koubi is strongly influenced by aquaculture activities, notably a fish farming training center and floating cages, which contribute to nutrient enrichment and reduced water transparency. The Tiébissou reservoir, a small artificial water body directly connected to Lake Koubi, is intensively used by the local population for market gardening.

These four sites illustrate a gradient of anthropogenic influence, ranging from near-natural to highly disturbed conditions (peri-urban, urban, aquaculture, and agricultural). This gradient provides a relevant framework for studying cyanobacterial communities and analyzing the environmental factors that influence their diversity and abundance in tropical aquatic ecosystems (Fig. 1).

### Sampling design and sample collection

The study was conducted from March 2023 to May 2024, focusing on four lakes representing distinct anthropogenic contexts. Monthly sampling spanned both main climatic seasons, dry and rainy, enabling the characterization of intra-seasonal variations in cyanobacterial communities. A total of nine sampling campaigns were carried out (March, April, May, June, July, October, and December 2023, and March to May 2024), providing adequate temporal coverage while minimizing the influence of extreme climatic events such as heavy rainfall or prolonged drought.

In each lake, three sampling stations were established to ensure optimal spatial representativeness. One station was located at the inlet of the main tributaries, which are major sources of nutrients and suspended solids. A second station was situated in the area used for drinking water abstraction, and a third targeted zones most directly affected by human activities, such as aquaculture cages, market gardens, or recreational areas in urban and agricultural lakes. For Lake Loka, which is minimally impacted by anthropogenic activities, the third station was positioned in the central pelagic zone. This spatial design accounted for anthropogenic pressure gradients while ensuring sample independence, in accordance with principles of experimental ecology (Hurlbert 1984).

Sampling was conducted between 6 a.m. and 9 a.m. at all stations. Water transparency was measured using a Secchi disk, and the sampling depth was defined as 2.5 times the Secchi depth to integrate the entire photic layer. Water samples were collected using an integrated water sampler, which was slowly lowered from the surface to the predetermined depth and subsequently raised to obtain a composite sample representative of the water column. This procedure was repeated as necessary to obtain the required volume. Samples were homogenized prior to subsampling for chlorophyll-a determination, nutrient analysis, and quantitative cyanobacterial enumeration.

For taxonomic identification (qualitative analysis), 10 L of water was collected using a bucket and concentrated by ten successive filtrations through a 20 µm mesh plankton net. Subsamples of 50 ml, intended for both qualitative and quantitative analyses, were transferred into sterile vials, immediately fixed with neutral formalin (final concentration: 5%), and stored until laboratory processing.

### Physicochemical analysis of water samples and chlorophyll-a measurement

Physicochemical parameters were measured in situ using a WiMo multiparameter probe (NKE Instrumentation, France). Prior to each sampling campaign, sensors for temperature, pH, turbidity, dissolved oxygen, and conductivity were manually installed and connected to a laptop via a Wi-Fi interface. This allowed verification of sensor operation, calibration, and acquisition settings in accordance with the manufacturer’s specifications.

At each station, water depth was initially estimated using a 20 cm Secchi disk, submerged on the shaded side of the boat to minimize light interference. The WiMo probe was then submerged approximately 30 cm below the surface to avoid sediment disturbance and surface turbulence. Measurements were recorded once stabilized, typically within 30–60 s, and automatically stored in the probe’s internal memory before transfer to a laptop for archiving and processing. After each measurement session, the probe and sensors were rinsed with fresh water, dried, and stored in a clean, dry environment, following the manufacturer’s recommendations to ensure measurement accuracy for subsequent campaigns.

For nutrient analyses, including nitrate, nitrite, ammonium, and orthophosphate, 500 ml subsamples were collected at each station, transported in insulated coolers with ice packs (Carboglass), and analyzed at the SODECI laboratory (Côte d’Ivoire).

Chlorophyll-a concentrations were determined following the AFNOR (NF T90-117 1999) standard, with minor adjustments for field conditions (Bamory et al. 2025). One liter of water per station was collected, stored in the dark at 4°C, and a 200 ml subsample was filtered through Whatman GF/F glass fiber filters (0.7 µm, 47 mm diameter) to concentrate phytoplankton biomass. Filters were immersed in 90% acetone for pigment extraction and stored in the dark at 4°C for 24 h. Samples were subsequently centrifuged at 2000 r/m for 15 min. Absorbance was measured at 665 nm and 750 nm before and after acidification with two drops of 0.1 N HCl. Chlorophyll-a concentrations were calculated according to Lorenzen (1967) and expressed in µg/L.


\begin{eqnarray*}
{\mathrm{chl\ a\ }}\left( {{\mathrm{\mu g\ }}/{\mathrm{\ L}}} \right) = {\mathrm{\ }}\frac{{26,7{\mathrm{\ X\ }}\left( {{\mathrm{E}}1 - {\mathrm{E}}2} \right){\mathrm{X\ V}}}}{{{\mathrm{l\ X\ V}}\mathcal{g}}}
\end{eqnarray*}

E1 = absorbance before acidification (DO665-DO750)0

E2 = absorbance after acidification (OD665-DO750)

V: volume of acetone (ml)

Vg: volume of filtered water

l: optical path length of the cell (cm)

### Identification and enumeration of cyanobacteria

Microscopic analyses were conducted using a Zeiss Motic BA310 optical microscope equipped with a digital imaging system. Cyanobacteria were identified to the genus level based on morphological characteristics, including cell shape and size, filamentous or colonial organization, and the presence or absence of heterocysts and/or akinetes. Taxonomic identification followed the classification keys of Komárek and Anagnostidis (1989); Komárek (2014), complemented by (Arif et al. 2023).

Enumeration of cyanobacteria was performed using a Malassez counting chamber in accordance with the NF EN 15204 standard (AFNOR 2006) and the methodological recommendations of Laplace-Treyture et al. (2009). The Malassez chamber consists of a calibrated grid defining a known volume, allowing direct calculation of cell densities. Prior to counting, samples were gently homogenized to ensure even distribution of biological units. An aliquot was then transferred into the counting chamber and allowed to settle for sufficient time to permit cell stabilization. A preliminary examination at low magnification (× 4 or × 10) was conducted to verify the homogeneous and random distribution of organisms. Counts were subsequently performed at × 40 magnification using the field-of-view method.

For each sample, at least 30 microscopic fields were examined to ensure statistical representativeness and minimize counting error. Counting units were defined according to the morphological organization of the observed cyanobacteria (individual cells, filaments, or colonies, depending on the taxon). Absolute abundance was expressed as individuals per liter (ind·L⁻¹). It was calculated from the mean number of counted units, taking into account the analyzed chamber volume and any applied dilution factor. Relative abundance was determined for each genus to assess its contribution to the overall cyanobacterial community structure. It was calculated as the percentage ratio of the absolute abundance of a given genus to the total absolute cyanobacterial abundance.

### Data analysis

All statistical analyses were performed using R software (version 4.4.1; R Development Core Team, 2025) with the *vegan, car*, and *ggplot2* packages (Oksanen et al. 2015). Residual normality was assessed using the Shapiro–Wilk test. As the data deviated from normality (*P* < .05), non-parametric tests were applied: the Kruskal–Wallis test was used to compare differences among stations, lakes, and seasons, followed, when appropriate, by post hoc pairwise comparisons using the Dunn test. When *P* > .05, a one-way ANOVA was performed with the appropriate post hoc test (Zar 1999). Differences were considered significant at *P* < .05, highly significant at *P* < .01, and very highly significant at *P* < .001.

Cyanobacterial diversity was quantified using the Shannon–Wiener index (H′), Pielou’s evenness index (J), and specific richness (S, total number of genera per sample). These indices were employed to compare community diversity and structure among lakes and across seasons.

Relationships between physicochemical parameters and cyanobacterial abundance, Shannon diversity, and chlorophyll-a concentration were assessed using Pearson correlation analyses.

Generalized linear models (GLMs) were constructed to identify environmental variables influencing cyanobacterial abundance, diversity, and biomass. Collinearity among explanatory variables was evaluated using the variance inflation factor (VIF; *car* package), and the final model was selected based on the lowest *R*² values and the Akaike information criterion (AIC) (Zuur et al. 2009).

Redundancy analyses (RDA) were conducted on cyanobacterial abundances and physicochemical parameters, with site and season included as covariates, using the rda () function of the *vegan* package. Non-metric multidimensional scaling (NMDS) was also performed by combining data from all four lakes, and spatial and temporal differences in community structure were assessed using PERMANOVA based on Bray–Curtis distances with 999 permutations (adonis2, *vegan*) (Anderson 2001).

## Results

### Lake physico-chemical properties and chlorophyll-a concentration

Analysis of the physicochemical properties of Lakes Kan, Koubi, Loka, and Tiébissou Reservoir revealed significant seasonal variability, while spatial variability within each lake was generally low. The only notable spatial variation was observed for turbidity in the Koubi aquaculture lake (*P* = .041), with higher values near agricultural activities and lower values at the catchment station, indicating a localized influence of diffuse inputs on water quality. For all other parameters and lakes, differences among sampling stations were not statistically significant (*P* > .05), reflecting relative intra-lake homogeneity (Supplementary Fig. S1). Seasonal variability, however, was more pronounced.

In the urban Lake Kan, water transparency tended to be higher during the dry season (152.3 ± 59.2 cm) compared with the rainy season (103.4 ± 9.6 cm), although this difference was not statistically significant (*P* = .242). Temperature remained stable across seasons (*P* = .534), whereas pH, nitrite, nitrate, orthophosphate, turbidity, and dissolved organic matter concentrations increased significantly during the rainy season (*P* < .05 to *P* < .001), indicating the influence of precipitation and runoff on water chemistry. Ammonium, conductivity, and dissolved oxygen did not show significant seasonal changes (*P* > .05).

In Koubi Lake, significant seasonal differences were observed for temperature (*P* < .05), orthophosphate (*P* < .05), ammonium (*P* < .01), dissolved oxygen (*P* < .05), and dissolved organic matter concentration (*P* < .01), likely reflecting seasonal effects of aquaculture practices and local hydrological conditions.

In Loka Lake, only orthophosphate (*P* < .01), nitrate (*P* < .05), and dissolved organic matter concentration (*P* < .001) increased significantly during the rainy season.

For Tiébissou Reservoir, which is used for agricultural purposes, temperature and pH were higher during the dry season (*P* < .05), while ammonium concentrations were higher during the rainy season (*P* < .05). No other parameters exhibited significant seasonal variation.

**Figure 2:**
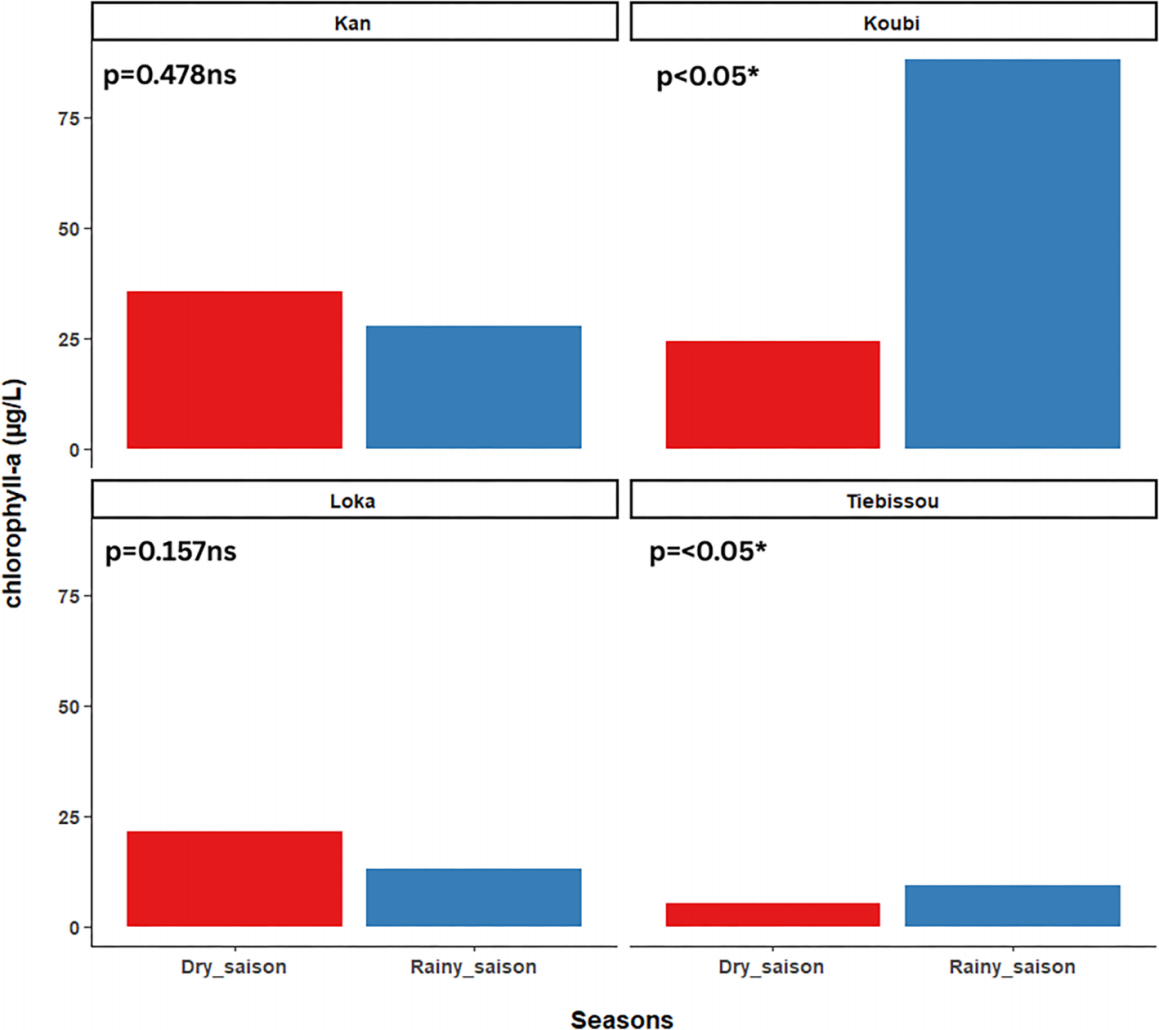
Variation in chlorophyll-a concentrations recorded in the different sampled lakes across seasons. Statistical significance levels are as follows: ns: *P* > .05 (not significant); ** *P* < .01 (highly significant); *** *P* < .001 (very highly significant).

Chlorophyll-a concentrations also exhibited both seasonal and spatial variation (Fig. 2). Significant spatial variation was detected only in Koubi Lake (*P* = .036), with higher concentrations near fish cages compared with other sampling stations, suggesting a local influence of aquaculture on phytoplankton production. Seasonally, chlorophyll-a concentrations increased significantly in the Koubi aquaculture lake and the Tiébissou agricultural reservoir during the rainy season (*P* < .05). Conversely, in urban Lake Kan and peri-urban Loka Lake, chlorophyll-a concentrations tended to be higher during the dry season; however, these seasonal differences were not statistically significant (*P* > .05) (Supplementary Fig. S1). (Table 1).

**Table 1:** Physico-chemical properties of Lakes Kan, Loka, and Koubi, as well as the Tiébissou Reservoir, and their significance statistics.

Lakes	Seasons	Transparency (cm)	Temperature (°C)	pH	Orthophosphate (mg/L)	Ammonium (mg/L)	Nitrite (mg/L)	Nitrate (mg/L)	Turbidity (NTU)	Conductivity (us/cm)	Dissolved oxygen (mg/L)
Kan	Dry	152.28 ± 59.24	24.3 ± 0.97	7.2 ± 0.15	0.2 ± 0.16	3.62 ± 2.28	0.01 ± 0.01	0.77 ± 0.83	337.39 ± 169.88	360.36 ± 33.81	2.55 ± 3.03
	Rainy	103.35 ± 9.55	27.62 ± 3.11	7.27 ± 0.78	1.35 ± 1.03	0.54 ± 0.59	0.11 ± 0.24	1.08 ± 0.9	16.49 ± 13.48	328.96 ± 116.51	1.4 ± 1.17
	*P*-value	.242 ns	.534 ns	< .01**	< .01**	.07 ns	< .05*	< .001***	< .001***	.52 ns	.15 ns
Koubi	Dry	101.6 ± 8.21	28.92 ± 0.32	6.56 ± 0.14	2.89 ± 1.5	4.26 ± 3.4	1.34 ± 1.52	0.4 ± 0.17	6.63 ± 4.51	88.98 ± 4.99	0.43 ± 0.09
	Rainy	103.35 ± 9.55	28.73 ± 2.68	8.18 ± 1.15	0.81 ± 1.12	4.26 ± 07.09	0.12 ± 0.18	1.31 ± 0.73	201.14 ± 313.63	93.06 ± 23.58	3.12 ± 2.6
	pvalue	.432 ns	< .05*	.628 ns	< .05*	< .01**	.759 ns	.073 ns	.30 ns	.77 ns	< .05*
Loka	Dry	141.21 ± 9.54	28.11 ± 3.8	7.22 ± 0.06	0.11 ± 0.11	1.1 ± 1.77	0.02 ± 0.01	0.78 ± 0.8	3.9 ± 1.95	58.75 ± 7.73	3.45 ± 3.31
	Rainy	158.48 ± 25.02	26.74 ± 5.23	7.51 ± 0.65	1.39 ± 1.32	0.87 ± 1	0.23 ± 0.46	1.68 ± 1.75	42.64 ± 117.04	113.86 ± 100.6	1.65 ± 1.67
	*P-*value	.28 ns	.952 ns	.557 ns	< .01**	.161 ns	.682 ns	< .05*	.43 ns	.19 ns	.07 ns
Tiébissou	Dry	140.27 ± 3.04	28.87 ± 0.63	7.11 ± 0.07	7.03 ± 8.76	7.75 ± 3.69	1.34 ± 1.15	0.34 ± 0.3	110.99 ± 160.91	82.03 ± 18.25	2.47 ± 0.15
	Rainy	127.68 ± 37.66	27.31 ± 2.66	7.55 ± 0.34	0.94 ± 1.52	1.64 ± 3.33	0.17 ± 0.63	1.65 ± 2.63	24.03 ± 63.59	105.36 ± 24.5	4.82 ± 2.13
	*P-*value	.064 ns	< .05*	< .05*	.393 ns	< .05*	.279 ns	.279 ns	.07 ns	.12 ns	.071 ns

Values represent means ± standard errors. Statistical significance levels are as follows: ns: *P* > .05 (not significant); ** *P* < .01 (highly significant); *** *P* < .001 (very highly significant).

### Composition of the cyanobacterial community

Based on morphological identification, 19 cyanobacterial genera were recorded across all studied lakes. Overall, the community was dominated by *Aphanocapsa* (24.36%), followed by *Pseudanabaena* (15.37%), and *Cylindrospermopsis* (11.72%). Less abundant genera included *Microcystis, Chroococcus, Gomphosphaeria, Aphanothece, Gleocapsa, Merismopedia, Neosynechococcus, Limnospira, Arthrospira, Limnolyngbya, Oscillatoria, Phormidium, Anabaemospsis, Anabaena, Leptolyngbya*, and *Lyngbya*.

**Figure 3:**
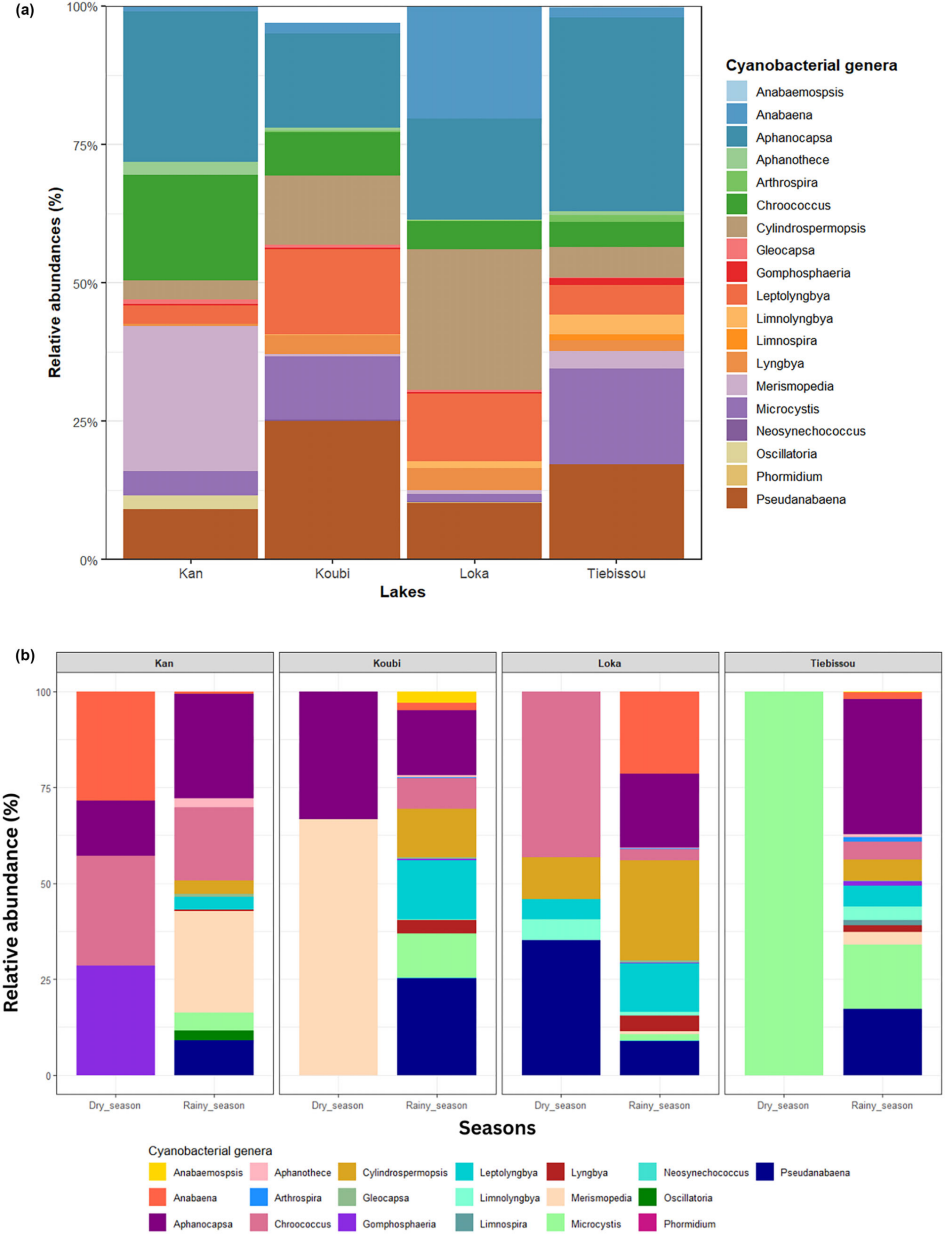
Relative abundances of cyanobacterial genera identified in the lakes of the different study areas. Relative abundances (%) represent the proportion of each genus in relation to the total number of genera in each lake. Relative abundances of cyanobacterial genera identified in the lakes of the different study areas during dry and rainy seasons.

The number of genera and their relative abundances varied among lakes, indicating marked spatial structuring of cyanobacterial communities (Fig. 3a). In urban Lake Kan, 13 genera were identified, with *Aphanocapsa* (27.17%), *Merismopedia* (26.2%), and *Chroococcus* (19.07%) being the most abundant. Koubi aquaculture lake hosted 16 genera, dominated by *Pseudanabaena* (25.08%), *Aphanocapsa* (17.04%), and *Leptolyngbya* (15.32%). Peri-urban Lake Loka exhibited the highest richness, with 17 genera, mainly *Cylindrospermopsis* (25.31%), *Anabaena* (20.22%), and *Aphanocapsa* (18.24%). In the agricultural Tiébissou Reservoir, 16 genera were recorded, dominated by *Aphanocapsa* (34.98%), *Microcystis* (17.27%), and *Pseudanabaena* (17.17%).

Significant seasonal variations were also observed in cyanobacterial community structure (Fig. 3b). In Lake Kan, the dry season was characterized by *Aphanocapsa* (14.28%), *Anabaena* (28.57%), *Chroococcus* (28.57%), and *Gomphosphaeria* (28.57%), whereas the rainy season was dominated by *Aphanocapsa* (27.34%), *Merismopedia* (26.56%), and *Chroococcus* (18.94%).

In Koubi Lake, the dry season community was dominated by *Merismopedia* (66.67%) and *Aphanocapsa* (33.33%), while the rainy season presented higher diversity, with *Pseudanabaena* (25.21%), *Aphanocapsa* (16.95%), and *Leptolyngbya* (15.40%) as the dominant genera.

In Tiébissou Reservoir, the dry season community was almost entirely dominated by *Microcystis* (99.98%), whereas in the rainy season, the structure became more balanced, with *Aphanocapsa* (35.21%), *Pseudanabaena* (17.28%), and *Microcystis* (16.73%) predominating.

Finally, in peri-urban Lake Loka, the dry season community was dominated by *Aphanocapsa* (19.25%), *Anabaena* (21.34%), and *Cylindrospermopsis* (26.12%). During the rainy season, the community exhibited a more balanced structure, with *Cylindrospermopsis* (26.11%), *Anabaena* (21.34%), *Aphanocapsa* (19.25%), and *Leptolyngbya* (12.53%) as the main genera.

### Abundance and diversity of cyanobacteria

Analysis of cyanobacterial communities revealed pronounced seasonal variations, both among lakes and within individual lakes. Overall, total absolute cyanobacterial abundance increased significantly during the rainy season across all lakes. During the dry season, abundances were lower and relatively homogeneous in all systems (Table 2). Spatially, total absolute abundances did not differ significantly among sampling stations (*P* > .05), although stations located near areas of human activity or aquaculture exhibited locally elevated values (Supplementary Fig. S2, Supplementary Table S1).

**Table 2:** Average total cyanobacterial abundance (Individuals. /L) by season in each surveyed lake.

Lakes	Seasons	Average total density (Individuals/L)	*P*-value
Kan	Dry	1.1 × 10^6^	< .001***
	Rainy	24 × 10^6^	
Koubi	Dry	2 × 10^6^ b	< .001***
	Rainy	55.3 × 10^6^ a	
Loka	Dry	6.1 × 10^6^ b	< .001***
	Rainy	31.9 × 10^6^ a	
Tiebissou	Dry	2 × 10^6^ b	< .001***
	Rainy	38.3 × 10^6^ a	

Statistical significance levels are as follows: *** *P* < .001 (very highly significant).

Assessment of alpha diversity, including Shannon–Wiener index (H′), and Pielou’s evenness index (J), also indicated intra- and inter-lake variations; however, these differences were not statistically significant (*P* > .05) (Supplementary Fig. S2, Supplementary Table S1). Lakes influenced by anthropogenic activities (aquaculture, agriculture, and urbanization) generally displayed moderate to high richness and diversity, with consistently high evenness. In contrast, the less impacted peri-urban Loka control lake exhibited pronounced spatial heterogeneity: some stations were sparsely colonized with low evenness (*J* = 0.62–0.63), while others harbored well-balanced communities (*J* > 0.9).

### Linking lake physico-chemical properties with cyanobacterial diversity and abundance

The Pearson correlation coefficients between abundance and Shannon alpha diversity, as well as between chlorophyll-a concentration and the lakes' physicochemical properties, are shown in the Table 3. Cyanobacterial abundance was negatively correlated with transparency (*r* = −0.10), nitrite (*r* = −0.16), and Conductivity (*r* = −0.10), although this correlation was not significant (*P* > .05). In contrast, abundance was significantly and positively correlated with pH (*r* = 0.57) and temperature (*r* = 0.23). Chlorophyll-a was negatively correlated with transparency (*r* = −0.20), nitrate (*r* = −0.08), nitrite (*r* = −0.06), orthophosphate (*r* = −0.09), and conductivity (*r* = −0.03). In contrast, pH (*r* = 0.25) and turbidity (*r* = 0.42) were positively and significantly correlated with chlorophyll-a. Shannon’s alpha diversity showed a significant negative relationship with turbidity (*r* = −0.32). Several negative, although non-significant, correlations were observed between Shannon alpha diversity and transparency, nitrate, pH, and dissolved oxygen. The diversity index was positively correlated with nitrite, ammonium, temperature, and conductivity.

**Table 3:** Pearson correlation coefficients linking lake physico-chemical properties with abundance, Shannon alpha diversity, chlorophyll-a.

Properties	Abundance	Chlorophyll-a	Shannon alpha diversity
Transparency	− 0.10	− 0.20*	− 0.03
Nitrate	0.02	− 0.08	− 0.10
Nitrite	− 0.16	− 0.06	0.08
Ammonium	0.02	0.16	0.05
Orthophosphate	− 0.13	− 0.09	0.11
pH	0.57***	0.25*	− 0.15
Temperature	0.23*	0.04	0.16
Turbididy	0.02	0.42***	− 0.32***
Conductivity	− 0.10	− 0.03	0.06
Dissolved_oxygen	0.09	0.01	− 0.08

Statistically significant correlation results are indicated by asterisks, and the thresholds for correlation strength classifications are as follows: * *P* < .05, moderate correlation; *** *P* < .01, strong correlation.

Analysis using (Generalized Linear Model) GLM revealed significant positive and negative relationships between cyanobacterial abundance and chemical properties (*P* < .05, Table 4). Cyanobacterial abundance increased in lakes with higher levels of transparency, nitrate, ammonium, pH, temperature, conductivity, and dissolved oxygen. High levels of nitrite, orthophosphate, and turbidity significantly reduced cyanobacterial abundance. The GLM indicated that the physicochemical properties of the lakes explained approximately 41.48% of the variation in cyanobacterial abundance (adjusted R² = 0.4148). The linear regression model further showed that chlorophyll-a concentration decreased with increasing levels of transparency, nitrate, ammonium, orthophosphate, conductivity, and dissolved oxygen. In contrast, chlorophyll-a concentration was positively associated with nitrite, pH, temperature, and turbidity. The model reveals that lake physico-chemical properties explain 3.7% of the variability in chlorophyll-a (R-adj = 0.037). An increase in turbidity level was associated with a significant reduction (*P* < .001) in alpha diversity (Shannon index). Alpha diversity (Shannon index) was negatively associated with nitrate, pH, turbidity, and dissolved oxygen levels.

**Table 4:** Influence of lake physico-chemical properties on abundance, shannon alpha diversity and Chlorophyll-a. The effects on abundance and shannon alpha diversity were highlighted using a GLM, while the effects on the chlorophyll-a were obtained using a simple linear regression model.

	Abundance		Chlorophyll-a		Shannon alpha diversity	
	Estimate+SE	*P*-value	Estimate+SE	*P*-value	Estimate+SE	*P*-value
(Intercept)	5.81 ± 0.007	< .001***	-28.18 ± 67.80	.678	0.745 ± 0.93	.425
Transparency	0.001 ± 0.000	< .001**	-0.369 ± 0.131	< .01**	0.001 ± 0.001	.476
Nitrate	0.16 ± 0,0006	< .001***	-3.10 ± 3.324	.353	-0.033 ± 0.04	.464
Nitrite	-0.495 ± 0.002	< .001**	8.19 ± 10.81	.450	0.094 ± 0.14	.524
Ammonium	0.037 ± 0.0001	< .001***	-0.104 ± 1.28	.935	0.028 ± 0.02	.107
Orthophosphate	-0.034 ± 0.0004	< .001**	-1.33 ± 2.430	.584	0.009 ± 0.03	.786
pH	0.38 ± 0,0004	< .001***	12.86 ± 5.728	.027*	-0.053 ± 0.07	.502
Temperature	0.048 ± 0.0001	< .001***	0.42 ± 1.216	.730	0.022 ± 0.01	.181
Turbididy	-0.0003 ± 0.000	< .001**	0.13 ± 0.025	< .001***	-0.001 ± 0.00	< .001***
Conductivity	0.0001 ± 0.000	< .001**	-0.02 ± 0.036	.486	0.0005 ± 0.00	.304
Dissolved_oxygen	0.005 ± 0.0002	< .001**	-1.56 ± 2.02	.442	-0.026 ± 0.02	.349

Values represent means±standard errors. Statistical significance levels are as follows: *statistically significant; **highly significant; ***very highly significant.

### Multivariate analyses of cyanobacterial community structure

#### Environmental drivers of community structure (RDA)

**Figure 4:**
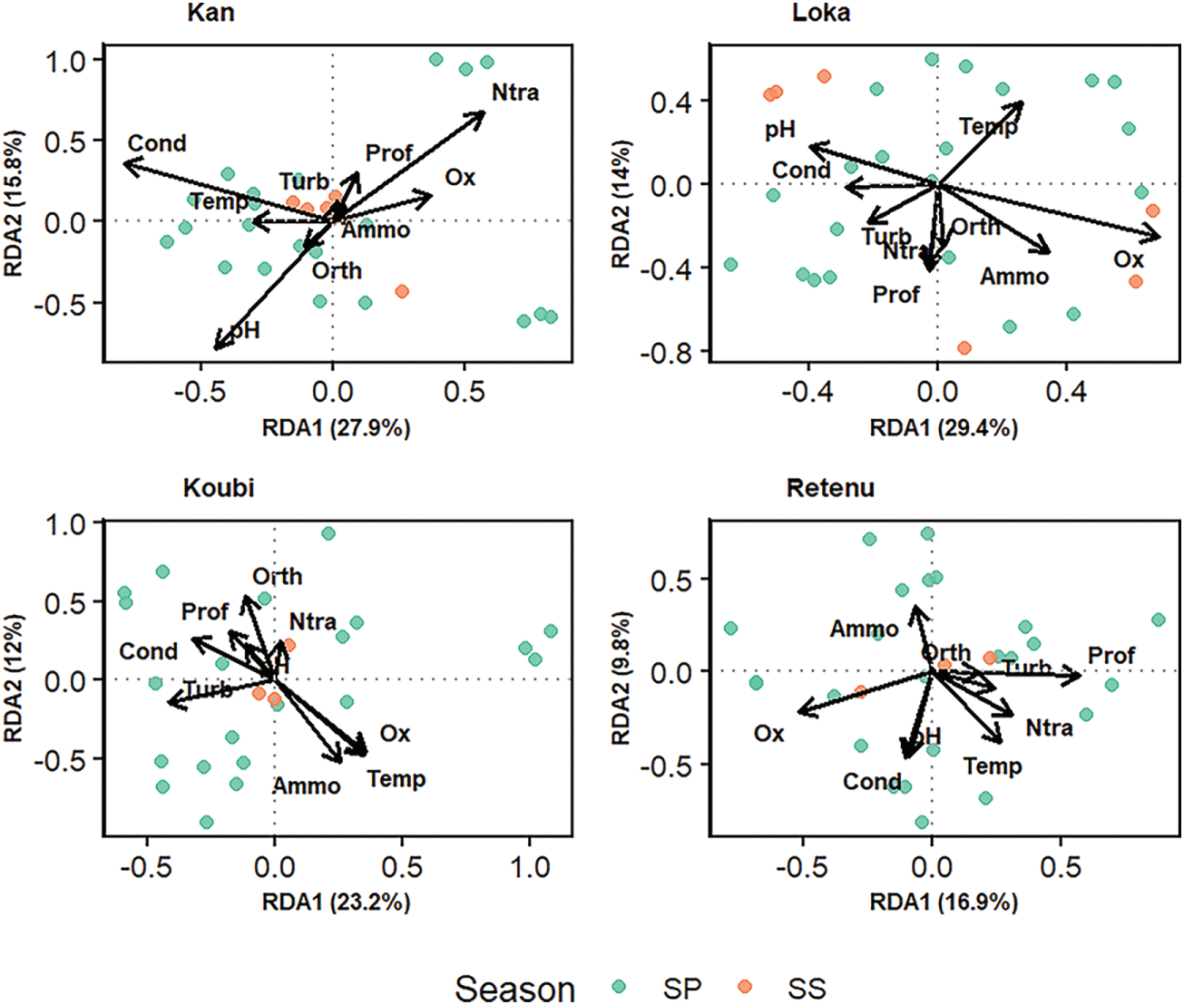
RDA ordinations performed separately for each lake to assess the influence of environmental variables on cyanobacterial community structure.

To assess the influence of environmental variables on cyanobacterial community structure, (Redundancy Analysis) RDAs were conducted independently for each lake. This site-based approach allowed the identification of environmental drivers specific to each ecological context. Explanatory variables included physicochemical parameters (temperature, pH, turbidity, conductivity, dissolved oxygen, depth) and nutrients (nitrates, ammonium, orthophosphate), while sampling stations and seasons were included as covariates (RDA function, *vegan* package, R v4.4.1) (Fig. 4). Statistical parameters of the models are summarized in Tables 5 and 6. Global permutation tests indicated significant relationships between environmental variables and cyanobacterial communities in all systems (*P* < .05).

**Table 5:** RDA results and variance partitioning of cyanobacterial community structure across the four studied lakes.

Parameters	Lake Kan	Lake Koubi	Lake Loka	Lake Retenu of Tiebissou
Eigenvalues				
Axis 1 (RDA1)	0.128	0.103	0.150	0.063
Axis 2 (RDA2)	0.073	0.053	0.072	0.037
Explained variance (%)				
Axis 1	27.9	23.2	29.4	16.9
Axis 2	15.8	12.0	14.0	9.8
Cumulative (Axes 1 & 2)	43.7	35.2	43.4	26.7
Significance tests (*P*-values)				
Global model	< 0.001***	< 0.05*	< 0.001***	< 0.05*
Axis 1	< 0.001***	0.115	< 0.001***	0.096
Axis 2	< 0.05*	0.607	< 0.05*	0.668

Statistical significance levels are as follows: *statistically significant; **highly significant; ***very highly significant.

**Table 6:** Results of RDA variable significance tests for each lake.

Lake	Variable	*F*	*P*-value
Kan	Conductivity (Cond)	6.999	.001***
Kan	pH	5.395	.001***
Kan	Dissolved oxygen (Ox)	2.644	.022*
Kan	Temperature (Temp)	2.359	.036*
Koubi	Turbidity (Turb)	2.587	.024*
Koubi	Nitrates (NO_3_-)	2.559	.025*
Loka	Dissolved oxygen (Ox)	7.507	.001***
Loka	pH	3.502	.006**
Loka	Temperature (Temp)	2.686	.015*
Loka	Ammonium (NH_4_+)	2.042	.042*
Retenu of Tiebissou	Dissolved oxygen (Ox)	3.085	.006**

Statistical significance levels are as follows: *statistically significant; **highly significant; ***very highly significant.

In urban Lake Kan, the RDA model explained 45.0% of the total variance, with an adjusted R² of 0.30 (*F* = 2.44; *P* = .001). The first canonical axis (RDA1) accounted for 27.9% of the variance (*P* = .001) and the second axis (RDA2) for 15.8% (*P* = .019), together explaining 43.7% of the variance for the first two axes. Conductivity (*P* = .001), pH (*P* = .001), temperature (*P* = .036), and dissolved oxygen (*P* = .022) were the variables most strongly influencing community structure.

Peri-urban Lake Loka exhibited the most robust environmental structuring, explaining 51.4% of the total variance (adjusted *R²* = 0.355; *F* = 2.57; *P* = .001). Both RDA axes were significant (RDA1: 29.4%, *P* = .001; RDA2: 14.0%, *P* = .014), together accounting for 43.4% of the variance. Key environmental drivers in this lake included dissolved oxygen (*P* = .001), pH (*P* = .006), temperature (*P* = .015), and ammonium (*P* = .042).

In Koubi aquaculture lake, the overall explained variance was 43.4% (adjusted *R²* = 0.187; *F* = 1.60; *P* = .035). However, the first two axes were not statistically significant (RDA1: *P* = .115; RDA2: *P* = .607) and together accounted for 35.2% of the variance. In this environment, turbidity (*P* = .024) and nitrate concentration (*P* = .025) were the most influential environmental variables.

Finally, in the Tiébissou agricultural reservoir, although the global RDA model was significant (adjusted *R²* = 0.15; *F* = 1.54; *P* = .021), environmental control appeared comparatively weaker, explaining 37.7% of the variance. Dissolved oxygen was the only significant factor identified (*P* = .006). The first two axes showed marginal significance (RDA1 ; *P* = .096; RDA2: *P* = .668) and together accounted for 26.7% of the variance explained.

#### Community similarity and differentiation among lakes (NMDS and PERMANOVA)

**Figure 5:**
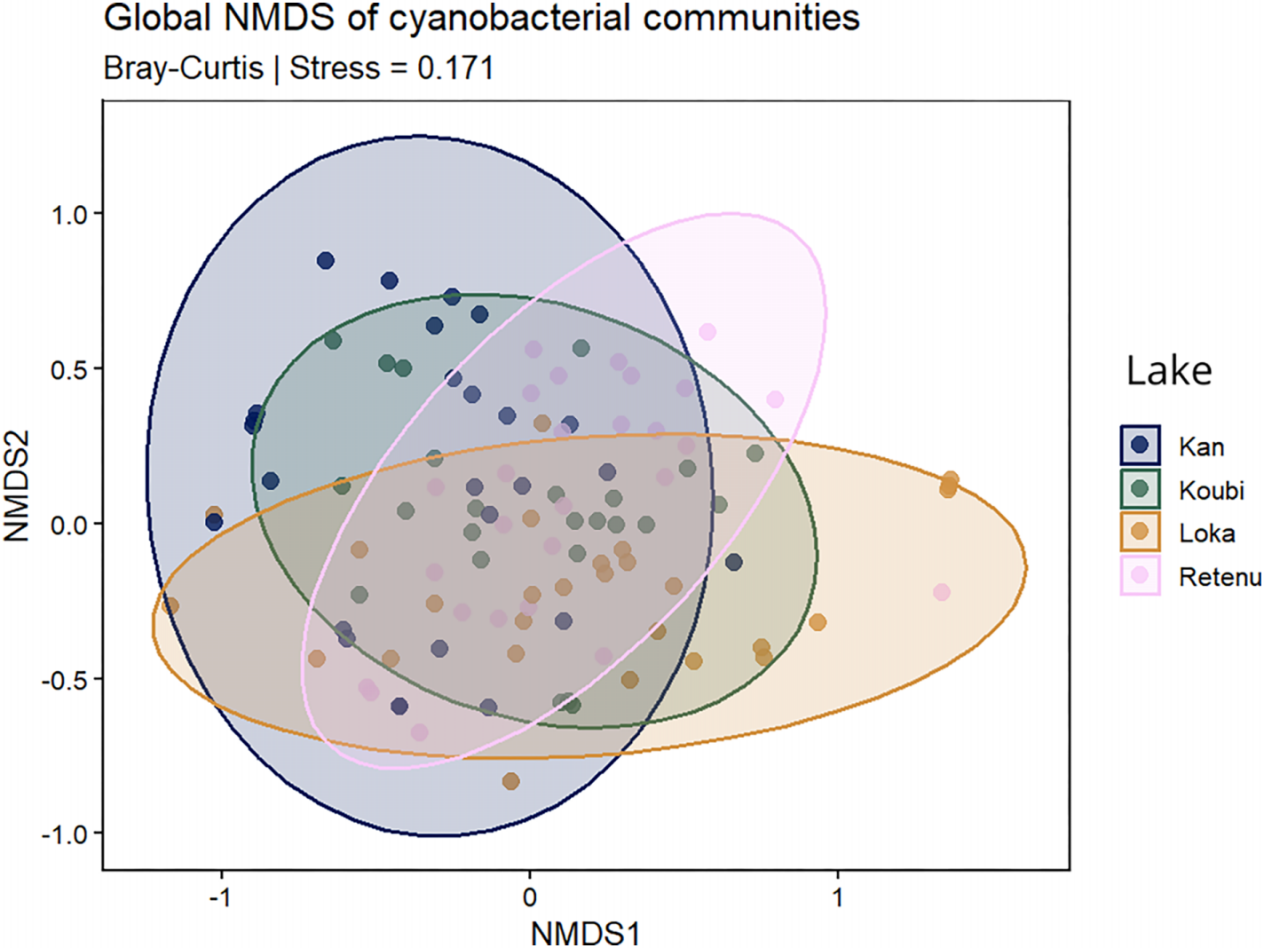
NMDS ordination based on Bray–Curtis dissimilarity, illustrating patterns of community similarity among the four lakes.

Cyanobacterial communities across the four lakes were compared using Non-metric Multidimensional Scaling (NMDS) based on absolute taxon abundance data, coupled with a PERMANOVA test. The NMDS ordination revealed substantial overlap among lake communities (stress = 0.171; Fig. 5), indicating a moderate level of compositional similarity across systems.

Nevertheless, the PERMANOVA analysis (adonis2, Bray–Curtis distance, 999 permutations) detected significant differences in community composition among lakes (*F* = 6.07; *R²* = 0.15; *P* = .001), demonstrating that despite the observed overlap in ordination space, lake identity significantly structured cyanobacterial assemblages.

## Discussion

This study demonstrates that the composition, abundance, and diversity of cyanobacterial communities vary significantly among lakes and across seasons, primarily driven by water physicochemical characteristics and associated anthropogenic pressures. The pronounced differences observed in chlorophyll-a concentrations further substantiate the strong influence of these environmental factors on phytoplankton biomass.

Overall, the investigated lakes exhibited pH values ranging from 6.55 to 8.17, reflecting slightly acidic to moderately alkaline conditions. Such pH levels are generally conducive to cyanobacterial proliferation, as previously reported (Kodandoor and Rajashekhar 2016). Water temperatures were consistently higher during the dry season (27.3–28.92°C) compared with the rainy season (24.29–28.72°C). Nevertheless, irrespective of season, temperatures remained relatively elevated, consistent with tropical aquatic systems, where mean water temperatures typically fluctuate around 29°C (Maberly et al. 2020).

Electrical conductivity displayed both spatial and seasonal variability. In contrast to the three other lakes, conductivity values in the urban Lake Kan were higher during the dry season. This pattern may be attributed to its densely urbanized setting, where evapoconcentration processes and sustained anthropogenic inputs increase mineral content during dry periods, while rainfall events promote dilution (Sulastri and Akhdiana 2022, Camara 2023). Seasonal variations in turbidity exhibited two contrasting patterns. In the peri-urban reference lake and the aquaculture lake (Loka and Koubi), turbidity values were higher during the rainy season. In contrast, the urban (Kan) and agricultural (Tiébissou Reservoir) lakes displayed elevated turbidity during the dry season. These findings suggest that turbidity dynamics are strongly modulated by land use. Peri-urban systems appear primarily influenced by diffuse inputs associated with precipitation events, whereas urban and agricultural lakes are more strongly shaped by sustained anthropogenic pressures and internal processes, particularly during the dry season (Nathalie et al. 2023).

Nutrient concentrations showed marked spatial variability among lakes. The aquaculture and agricultural systems (Koubi and Tiébissou Reservoir), which are hydrologically connected, exhibited elevated concentrations of ammonium and orthophosphate, whereas Loka recorded the highest nitrate levels. The enrichment in ammonium and orthophosphate observed in aquaculture and agricultural lakes likely reflects direct anthropogenic inputs combined with enhanced mineralization under frequently reducing conditions (Idowu et al. 2013, Kashindye et al. 2015). These results are consistent with previous studies reporting that aquaculture activities are commonly associated with increased ammonium and orthophosphate concentrations in aquatic ecosystems, primarily due to the decomposition of fish excreta and uneaten feed (Kashindye et al. 2015, Camara 2023), Likewise, agricultural runoff enriched in fertilizers contributes substantial nitrogen and phosphorus loads, thereby accelerating eutrophication processes in exploited lakes and waterways (Mezzacapo 2018).

Conversely, the predominance of nitrate in the peri-urban lake suggests a biogeochemical functioning more closely aligned with relatively undisturbed conditions. In well-oxygenated environments, ammonium is rapidly oxidized through nitrification, generally resulting in the dominance of NO₃⁻ among dissolved inorganic nitrogen forms (Baumann et al. 2022, Klotz et al. 2022).

Cyanobacterial community composition differed markedly among sites in relation to the intensity of anthropogenic pressures. The most disturbed ecosystems were characterized by a pronounced dominance of opportunistic and cosmopolitan taxa, notably *Aphanocapsa, Pseudanabaena*, and *Microcystis*. According to previous studies, these genera exhibit high bloom-forming potential and frequently co-occur in meso-eutrophic to eutrophic systems, often alongside taxa adapted to intermediate nutrient gradients (Hu et al. 2021). The predominance of *Aphanocapsa, Pseudanabaena*, and *Microcystis* in the most heavily anthropized sites thus reinforces the strong linkage between land use intensity and water quality degradation.

These findings are consistent with observations from temperate lakes in Chile, where Chile showed a clear ecological shift from diatom (Bacillariophyta) dominance in oligotrophic environments to cyanobacterial prevalence under meso-eutrophic and eutrophic conditions (Almanza et al. 2019). Such transitions illustrate the broader pattern of phytoplankton community restructuring along nutrient enrichment gradients.

In contrast, the dominance of *Cylindrospermopsis* and *Anabaena* in the peri-urban reference lake may be attributable to the high ecological plasticity of these filamentous genera. Their functional traits, including physiological flexibility and adaptive responses to fluctuating environmental conditions, enable persistence across a wide spectrum of ecological contexts, from relatively low nutrient availability to variable hydrodynamic regimes. Although frequently associated with eutrophic systems, several studies indicate that certain strains tolerate comparatively low nitrogen and phosphorus concentrations (Kapkov et al. 2019, Zheng et al. 2023).

Moreover, the coexistence of multiple ecotypes arising from microevolutionary processes may further explain their presence in less disturbed environments. In particular, some strains of *Cylindrospermopsis* have been shown to persist in well-oxygenated and moderately nutrient-limited systems, highlighting the adaptive potential of this genus (Pagni et al. 2020).

The correlation analyses and Generalized Linear Model (GLMs) underscore the pivotal role of physicochemical parameters in shaping cyanobacterial abundance and overall phytoplankton biomass. Both temperature and pH were positively associated with cyanobacterial abundance, consistent with their known preference for warm and slightly alkaline conditions (Paerl et al. 2011).

Cyanobacterial diversity, as measured by the Shannon index, was positively influenced by temperature and certain reduced nitrogen forms (NH₄⁺, NO₂⁻), suggesting that moderate nutrient enrichment can enhance niche heterogeneity (Salmaso et al. 2024). However, GLM results indicated a decline in alpha diversity in response to elevated turbidity and high pH, likely reflecting bloom events where dominant taxa competitively exclude less resilient species (Xiao et al. 2018, Panou and Gkelis 2022).

Total phytoplankton biomass, expressed as chlorophyll-a, was positively correlated with pH and turbidity, indicative of elevated primary production (Mageswaran and Ponnusamy 2025). Conversely, the negative relationships observed between chlorophyll-*a* and dissolved inorganic nutrients may reflect rapid nutrient assimilation during active phytoplankton growth, a mechanism commonly reported in productive aquatic ecosystems (Reynolds 2006, Garnier 2025). Similarly, the inverse association between dissolved oxygen concentrations and phytoplankton biomass could be attributed to enhanced community respiration driven by the decomposition of organic matter, which in some eutrophic systems may exceed gross primary production (Cheve and Lejolivet 2022, Guéguen et al. 2024).

RDA revealed that cyanobacterial communities exhibit differentiated responses to environmental variables, with each lake being associated with a specific set of structuring factors. In certain lakes, RDA models explained 45% of the variance with significant axes (*P* < .05), indicating that the measured environmental variables effectively capture the primary gradients structuring cyanobacterial assemblages. In contrast, aquaculture and agricultural lakes exhibited weaker models, with non-significant axes and lower explained variance (35.2% and 26.9%, respectively), suggesting a more complex or diffuse ecological structuring.

In the aquaculture lake, intermittent feed inputs and fluctuations in fish biomass likely generate substantial temporal variability, reducing the consistency of relationships between environmental parameters and biological communities (Lazard 2017, Girard 2024). Similarly, in the agricultural lake, diffuse inputs of nutrients and sediments from runoff can create spatially and temporally heterogeneous conditions (Cakir 2020). Furthermore, the absence of key trophic variables, such as total phosphorus and total nitrogen, may have constrained the explanatory power of RDA models in these systems, as these parameters strongly influence cyanobacterial dominance, species composition, and population dynamics (Boulkhiout Khaoula 2023, El Hadji Mamane 2025).

Variable significance tests indicated that dissolved oxygen was the most consistently influential factor, significant in urban, peri-urban, and agricultural lakes, suggesting its role as a major environmental gradient structuring cyanobacterial communities. In agricultural systems, where dissolved oxygen was the only significant variable, community structuring may primarily reflect internal metabolic processes, such as the balance between primary production and respiration, rather than external trophic gradients. This observation aligns with previous studies emphasizing the importance of oxygen in regulating metabolism and microbial diversity in aquatic environments (Cui et al. 2023, Fusi et al. 2023).

In the urban lake Kan, cyanobacterial composition was significantly influenced by pH and conductivity, highlighting the strong role of mineralization and acid–base conditions in structuring these communities. These findings are consistent with reports that pH and conductivity are key determinants of phytoplankton assemblages in disturbed urban lakes (Silva et al. 2011, Cui et al. 2023, Melese and Debella 2024, Zhao et al. 2024). In such environments, variability in mineralization, often exacerbated by runoff, can act as an environmental filter favoring taxa with efficient osmotic adaptation mechanisms while excluding more sensitive species (Souza et al. 2025).

In the Koubi aquaculture lake, turbidity and nitrate concentration were the only significant variables, suggesting that cyanobacterial dynamics in this system are mainly controlled by nitrogen availability and light limitation. Previous studies similarly indicate that the interaction between nutrient availability and light conditions is a key determinant of bacterial taxon dominance (Peter and Sommaruga 2016, Woodhouse et al. 2016). The significance of turbidity may also reflect selective pressure favoring shade-tolerant genera (e.g. *Planktothrix*) or those capable of buoyancy regulation (Reynolds et al. 2002, Tiwari et al. 2025).

Unlike heavily anthropized systems dominated by mineralization, the peri-urban reference lake exhibited a community structure largely governed by natural biological cycles. In this system, the combined influence of temperature, pH, and dissolved oxygen likely reflects the predominant role of internal biological processes in shaping cyanobacterial assemblages (Lu et al. 2013). Furthermore, the strong preference of cyanobacteria for ammonium as a nitrogen source, as reported by Le et al. 2024), may explain why this variable emerges as a significant driver of community structure in this environment.

NMDS and PERMANOVA analyses revealed partial overlap of cyanobacterial communities among the four lakes, yet significant overall differences were detected. These results indicate that, although some taxa are shared between systems, each lake harbors a distinct community. The observed similarities may be attributed to the geographical proximity of the four water bodies, all situated in the central region of the country, as well as the hydrological connectivity between the agricultural and aquaculture lakes, which could facilitate dispersal of certain taxa and contribute to partial community convergence. Nevertheless, the uniqueness of the communities in each lake underscores the predominant role of local environmental gradients, particularly turbidity, dissolved oxygen, and nutrient concentrations in shaping cyanobacterial assemblages and determining species composition (Xie et al. 2011, Liu et al. 2019).

Although cyanobacterial characterization in this study was based exclusively on morphological criteria, this approach remains widely used and ecologically meaningful, especially in tropical systems where blooms are frequent and often dominated by morphologically well-defined taxa. Morphological identification provides a direct assessment of bloom potential and associated ecotoxicological risks.

Future integration of molecular approaches, such as 16S rRNA gene sequencing or cyanobacteria-specific genetic markers, would provide enhanced taxonomic resolution, allowing detection of cryptic diversity and more detailed analysis of community dynamics. Such a combined approach would be particularly valuable in tropical ecosystems, which remain underrepresented in large-scale molecular surveys.

Overall, the complementary application of RDA, GLM, and NMDS analyses elucidates the ecological mechanisms structuring cyanobacterial communities and offers practical guidance for the management and restoration of tropical lakes by identifying and targeting environmental factors that promote cyanobacterial proliferation.

## Conclusion

The structure of cyanobacterial communities across different lakes is strongly influenced by the intensity of anthropogenic pressures and the associated physicochemical gradients. Highly disturbed systems are dominated by opportunistic genera capable of tolerating eutrophic and fluctuating conditions, whereas less impacted lakes exhibit more pronounced environmental structuring and potentially more balanced community compositions.

The pronounced sensitivity of cyanobacterial communities to physicochemical variables indicates that these ecosystems can respond rapidly to environmental changes, particularly those linked to urbanization, agriculture, and aquaculture activities. GLMs and correlation analyses revealed that ammonium, temperature, and conductivity positively affect cyanobacterial abundance and alpha diversity, whereas nitrites and turbidity exert negative effects. Total phytoplankton biomass (chlorophyll-a) decreases with transparency, nutrient concentrations (ammonium, orthophosphate, nitrate), and dissolved oxygen, but increases with pH, temperature, and turbidity.

RDA identified dissolved oxygen, pH, and temperature as the most recurrent explanatory variables, suggesting that oxygenation and thermal conditions play central roles in structuring cyanobacterial communities. However, the variability in the proportion of explained variance among lakes highlights that structuring mechanisms differ according to ecological context and watershed use. NMDS ordination showed a high overall similarity among cyanobacterial communities across the four lakes, while PERMANOVA confirmed significant differences between individual lake communities. These patterns reflect the interplay between regional taxa dispersal and local environmental constraints specific to each system.

The environmental drivers identified in these tropical lakes largely correspond to those reported for temperate systems, suggesting that the fundamental mechanisms governing cyanobacterial community assembly, such as environmental filtering and ecological selection, are broadly conserved across climatic zones. Nevertheless, the relative importance of individual variables varies according to lake location and the type of anthropogenic pressure.

Overall, these findings provide a robust scientific basis for the management of lakes under increasing anthropogenic pressure, highlighting key avenues for the conservation, restoration, and sustainable management of tropical freshwater ecosystems.

## Supplementary Material

fiag035_Supplemental_Files

## Data Availability

The datasets generated and/or analyzed during the current study are available from the corresponding author on reasonable request.
