# Supplementary material for: Ecological assessment of physico-chemical factors influencing the diversity and abundance of cyanobacteria in lakes of Côte d’Ivoire (Kan, Koubi, Loka, and Tiebissou)
Source: FEMS Microbiol Ecol. 2026 Mar 31;102(5):fiag035. doi: 10.1093/femsec/fiag035 (PMC13098367; doi:10.1093/femsec/fiag035)
Supplement: fiag035_Supplemental_Files [file fiag035_supplemental_files.zip › Tab_S1_.docx]

**Tab. S1**. Results of spatial variability tests for physicochemical parameters.

| **Lakes** | **Variables** | **Shapiro_p** | **Test** | **p_value** |
| --- | --- | --- | --- | --- |
| Kan | Temperature | 0,27538652 | ANOVA | 0,363 |
| Koubi | Temperature | 0,02539394 | Kruskal-Wallis | 0,773 |
| Loka | Temperature | 0,00766881 | Kruskal-Wallis | 0,888 |
| Tiebissou | Temperature | 0,0013947 | Kruskal-Wallis | 0,918 |
| Kan | pH | 2,5555E-05 | Kruskal-Wallis | 0,408 |
| Koubi | pH | 0,00085825 | Kruskal-Wallis | 0,977 |
| Loka | pH | 0,00340906 | Kruskal-Wallis | 0,909 |
| Tiebissou | pH | 0,01331767 | Kruskal-Wallis | 0,972 |
| Kan | Turbidity | 9,8274E-08 | Kruskal-Wallis | 0,988 |
| Koubi | Turbidity | 2,1735E-06 | Kruskal-Wallis | 0,843 |
| Loka | Turbidity | 3,8655E-10 | Kruskal-Wallis | 0,238 |
| Tiebissou | Turbidity | 7,2107E-10 | Kruskal-Wallis | 0,0416* |
| Kan | Conductivity | 3,9472E-07 | Kruskal-Wallis | 0,95 |
| Koubi | Conductivity | 0,00116732 | Kruskal-Wallis | 0,794 |
| Loka | Conductivity | 4,1515E-08 | Kruskal-Wallis | 0,702 |
| Tiebissou | Conductivity | 0,01032689 | Kruskal-Wallis | 0,491 |
| Kan | Dissolved oxygen | 6,3331E-05 | Kruskal-Wallis | 0,993 |
| Koubi | Dissolved oxygen | 0,00144257 | Kruskal-Wallis | 0,927 |
| Loka | Dissolved oxygen | 0,00039146 | Kruskal-Wallis | 0,979 |
| Tiebissou | Dissolved oxygen | 0,09723767 | ANOVA | 0,979 |
| Kan | depth | 6,9221E-08 | Kruskal-Wallis | 0,229 |
| Koubi | depth | 0,00065016 | Kruskal-Wallis | 0,708 |
| Loka | depth | 0,00017387 | Kruskal-Wallis | 0,387 |
| Tiebissou | depth | 7,6977E-06 | Kruskal-Wallis | 0,937 |
| Kan | Nitrate | 0,00203985 | Kruskal-Wallis | 0,857 |
| Koubi | Nitrate | 0,2863603 | ANOVA | 0,916 |
| Loka | Nitrate | 9,058E-07 | Kruskal-Wallis | 0,84 |
| Tiebissou | Nitrate | 1,9627E-09 | Kruskal-Wallis | 0,86 |
| Kan | Ammonium | 1,0135E-06 | Kruskal-Wallis | 0,74 |
| Koubi | Ammonium | 1,9047E-06 | Kruskal-Wallis | 0,807 |
| Loka | Ammonium | 9,5114E-07 | Kruskal-Wallis | 0,364 |
| Tiebissou | Ammonium | 1,6949E-07 | Kruskal-Wallis | 0,545 |
| Kan | Orthophosphate | 0,00075907 | Kruskal-Wallis | 0,753 |
| Koubi | Orthophosphate | 3,9382E-05 | Kruskal-Wallis | 0,982 |
| Loka | Orthophosphate | 2,9277E-05 | Kruskal-Wallis | 0,737 |
| Tiebissou | Orthophosphate | 7,2124E-09 | Kruskal-Wallis | 0,898 |
