# Supplementary material for: Ecological assessment of physico-chemical factors influencing the diversity and abundance of cyanobacteria in lakes of Côte d’Ivoire (Kan, Koubi, Loka, and Tiebissou)
Source: FEMS Microbiol Ecol. 2026 Mar 31;102(5):fiag035. doi: 10.1093/femsec/fiag035 (PMC13098367; doi:10.1093/femsec/fiag035)
Supplement: fiag035_Supplemental_Files [file fiag035_supplemental_files.zip › Tab_S2.docx]

Table S2:Relative abundances of cyanobacterial genera identified in the lakes of the different study areas. Relative abundances (%) represent the proportion of each genus in relation to the total number of genera in each lake

| Lacs | Kan | Koubi | Loka | Tiebissou |
| --- | --- | --- | --- | --- |
| Anabaemospsis (%) | 0.00 | 3.00 | 0.14 | 0.22 |
| Anabaena (%) | 0.96 | 1.97 | 20.23 | 1.84 |
| Aphanocapsa (%) | 27.17 | 17.04 | 18.25 | 34.99 |
| Aphanothece (%) | 2.31 | 0.51 | 0.14 | 0.76 |
| Arthrospira (%) | 0.00 | 0.26 | 0.14 | 1.19 |
| Chroococcus (%) | 19.08 | 7.79 | 5.09 | 4.54 |
| Cylindrospermopsis (%) | 3.47 | 12.59 | 25.32 | 5.51 |
| Gleocapsa (%) | 0.77 | 0.51 | 0.42 | 0.11 |
| Gomphosphaeria (%) | 0.39 | 0.26 | 0.28 | 1.19 |
| Leptolyngbya (%) | 3.28 | 15.33 | 12.16 | 5.40 |
| Limnolyngbya (%) | 0.00 | 0.26 | 1.27 | 3.56 |
| Limnospira (%) | 0.00 | 0.00 | 0.00 | 1.19 |
| Lyngbya (%) | 0.39 | 3.42 | 3.96 | 1.84 |
| Merismopedia (%) | 26.20 | 0.34 | 0.71 | 3.24 |
| Microcystis (%) | 4.43 | 11.47 | 1.41 | 17.28 |
| Neosynechococcus (%) | 0.00 | 0.17 | 0.14 | 0.00 |
| Oscillatoria (%) | 2.50 | 0.00 | 0.00 | 0.00 |
| Phormidium (%) | 0.00 | 0.00 | 0.14 | 0.00 |
| Pseudanabaena (%) | 9.06 | 25.09 | 10.18 | 17.17 |
