# Supplementary figures and images for: Ecological assessment of physico-chemical factors influencing the diversity and abundance of cyanobacteria in lakes of Côte d’Ivoire (Kan, Koubi, Loka, and Tiebissou)
Source: FEMS Microbiol Ecol. 2026 Mar 31;102(5):fiag035. doi: 10.1093/femsec/fiag035 (PMC13098367; doi:10.1093/femsec/fiag035)

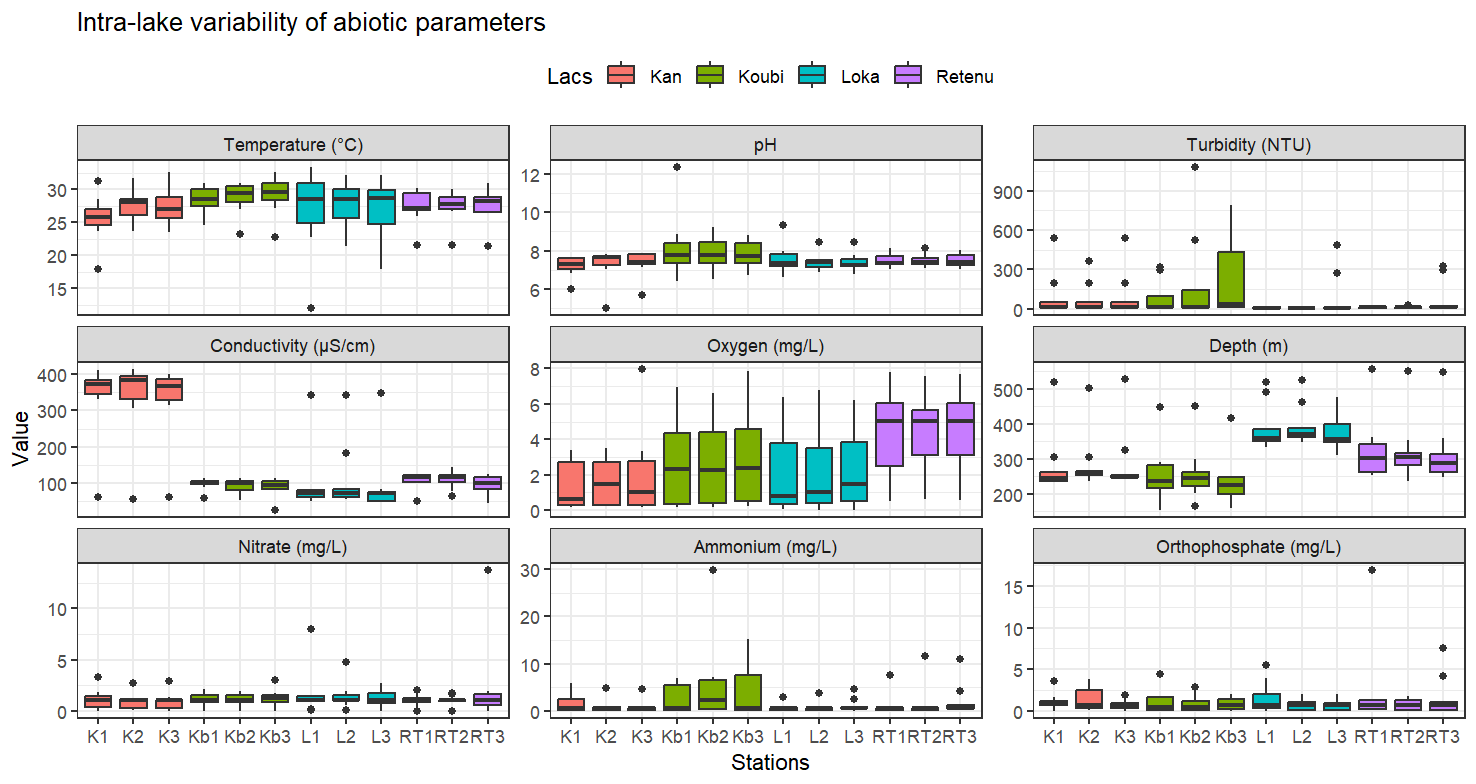

Supplement: fiag035_Supplemental_Files [file fiag035_supplemental_files.zip › Fig_S1_.png]

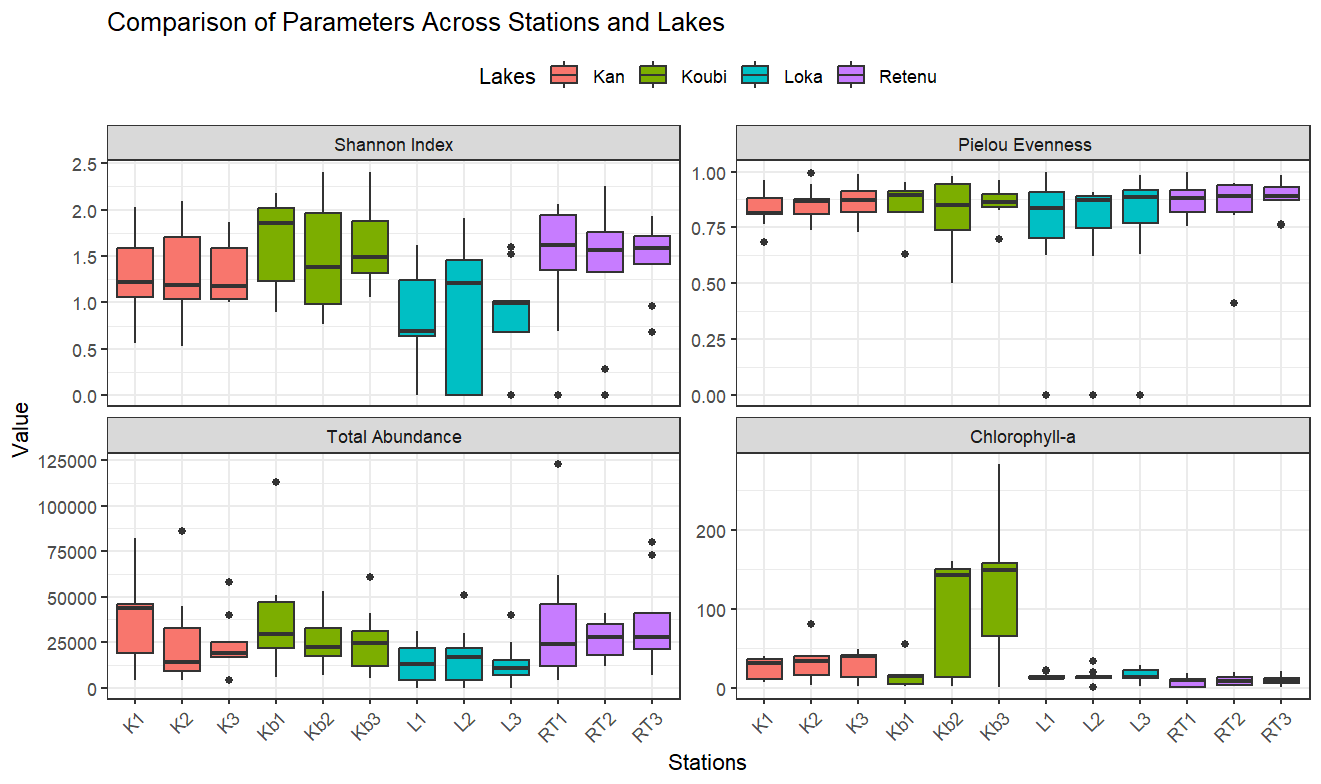

Supplement: fiag035_Supplemental_Files [file fiag035_supplemental_files.zip › Fig_S2_.png]
